# Feasibility and acceptability of “active” classroom workstations among French university students and lecturers: a pilot study

**DOI:** 10.1186/s12889-021-11074-3

**Published:** 2021-05-27

**Authors:** Sidney Grosprêtre, Gael Ennequin, Sophie Peseux, Laurie Isacco

**Affiliations:** 1 EA4660, C3S Culture Sport Health Society, UPFR Sports, 31, Chemin de l’Epitaphe, 25000 Besançon, France; 2 Université de Bourgogne-Franche-Comté, Besançon, France; 3 Université Clermont Auvergne, CRNH, AME2P, F-63000 Clermont-Ferrand, France; 4 EA3920 Prognostic Markers and Regulatory Factors of Cardiovascular Diseases and Exercise Performance Health Innovation (EPSI) platform, Univ. Bourgogne Franche-Comte, Besançon, France

**Keywords:** Sedentary lifestyle, Standing desk, Cycling desk, Classroom, College

## Abstract

**Background:**

Sedentary lifestyles plague today’s society in terms of physical, psychosocial and cognitive health. Students are particularly at risk because they spend most of their daily time sitting and inactive. The current pedagogical model must be rethought in order to promote students’ health, well-being and therefore their success. The objective of this project was to equip one classroom of the sport science department at a university with various active workstations (standing desks, Swiss balls, cycling desks, pedal- or stepper-board) and to evaluate the feasibility and influence of these types of active workstations on the cognitive and psychological factors of the students, and to collect the feedback of lecturers during this experience.

**Methods:**

Participation in the study was voluntary and students had the opportunity to choose or not to use an active workstation during lectures. A total of 663 students and 14 lecturers completed a survey to evaluate subjective feelings about physical, psycho-cognitive and academic aspects after their first experience with active workstations in the classroom.

**Results:**

The majority of students and lecturers reported positive effects of active workstations in reducing fatigue, distraction, and boredom. The preferred active workstations were the cycling desks and the Swiss balls. Finally, 89.4% of students favoured using active workstations in future lectures if they had the option, as well as 71% of lecturers, demonstrating the acceptance of such active workstations.

**Conclusion:**

The present study brings promising results toward a more general implementation of active workstations in universities. Once a familiarisation period is observed, having such workstations in the classroom could help prevent the deleterious effect of sedentary behaviour and promote a more active daily life for the future.

## Background

Student success is one of the university’s main challenges. It is mainly expressed through academic results and precise success criteria (e.g., percentage of admitted students, employability) and is only possible through the student’s general well-being. Promoting the physical and mental health of students is, therefore, a paramount aspect to be developed and maintained. As stated by the World Health Organisation, *“Health is a state of complete physical, mental and social well-being and not merely the absence of disease or infirmity”* [1]*.* This definition illustrates an holistic model, taking into account different components of health. Improving students’ life habits, such as quality of sleep and diet, physical activity, prevention of addictions, and reduction of stress appears crucial. However, due to our current lifestyles, these habits are negatively affected. University students, because of inherent consequences related to their status (e.g. distance from the parental home, limited financial resources, work, exam stress), are a particularly at-risk group [2]. This influences their general health and well-being and, therefore, their success and fulfilment.

Although proposing practical health initiatives throughout the students’ curriculum in order to promote their personal fulfilment (e.g., sport activities outside of lectures, balanced menus at the refectory), the university and, more generally, our current pedagogical model may unintentionally compromise these health actions. Indeed, sedentary behaviours (i.e. activities that do not increase energy expenditure substantially above the resting level, namely < 1.6 Metabolic Equivalent of Task [3]) are a feature of our current society with dramatic consequences in terms of health. Indeed, many studies have highlighted the positive association between the time spent in sedentary activities and the risks of all-cause mortality, some cancer occurrence and cardiometabolic morbidity, including cardiovascular diseases and type 2 diabetes [4–7]. Also, maintaining a seated position for several hours in a day, regardless of a person’s level of physical activity, constitutes a real health hazard, and many scientists have therefore noted the importance of regularly interrupting this position even for the most active or sporty people [8–10]. In addition, sedentary behaviours can be associated with increased boredom and decreased alertness, memory, concentration, motivation, and sleep quality. All of these are associated with reduced academic and social abilities [11–15].

It is recognised that, due to the nature of our professional and leisure activities, we spend more and more time in a seated position (e.g., TV, computer, reading, transport). Research studies highlight that this situation is particularly characteristic for young people and university students (e.g. lectures, lunches, public transports, revisions) [16–19]. Studies reported that university students spend on average 7 to 8 h a day in a sitting position (sedentary time being assessed either with questionnaires or accelerometers), suggesting that this population, due to their sedentary lifestyle, is at high risk of overall morbi-mortality [20–22]. Yet evidence suggests that regularly breaking up prolonged bouts of sedentary times (getting up, fidgeting, carrying out low-intensity activities) is beneficial for physical, psychological and cognitive health and attenuates the negative health consequences of sedentary time [23]. Thus, effective and sustainable interventions need to be promoted in order to counteract the health burden of excessive sedentary behaviours. In this regard, ecological models with active workstations in professional or school environments have been developed and appear as effective solutions. In a recent systematic review, active desks have been reported to be a promising tool to decrease sedentary behaviours in children and adolescents [24]. Similarly, sit-stand desks seem effective at reducing sitting time in children [25] and office workers [26]. While scientific studies on active workstations are increasingly numerous in the professional environment, those targeting students remain fragmented. In a study published in 2017, Jerome et al. proposed desks that allow standing and sitting for 6 weeks to nearly 500 students at a Midwestern university, USA [18]. Their results showed that the use of these desks increased the time spent standing during classes. At the same time, more than half of the students noted an increase in attention and a decrease in restlessness during class. More than a third noted an improvement in engagement and a reduction in boredom and fatigue. Similarly, a Canadian study showed the feasibility of implementing standing or pedal-operated workstations in university libraries [27], while Benzo and colleagues reported the overall acceptability of university students and lecturers of introducing standing desks in classrooms [16].

However, to our knowledge, no study has been conducted on the implementation of a variety of active workstations in French universities. By equipping a classroom with various active workstations and having the students and lecturers fill out a survey after a lecture, this study aimed at testing the feasibility and acceptability of such equipment. The survey aimed at questioning the impact of such active workstations on subjective markers of fatigue, attention and concentration compared to traditional workstations (usual chairs and desks), from students’ as well as from lecturers’ point of view. Ultimately, it was hypothesised that students and lecturers would accept the implementation of active workstations in the classroom and would be in favour of using such options in future lectures.

## Method

In September 2019, a classroom was equipped with active workstations at the sport department of the university of Franche-Comte in Besançon, France. Lectures in this room primarily involved first-year students (~ 18–20 years old). At the beginning of the academic year (September 2019), all students had been informed by their academic supervisor about the implementation and the development of active workstations in a classroom of the department. When students had lectures in this classroom, they were free to use the active workstations or not. Traditional workstations, i.e., “traditional” chairs and desks, were still present in sufficient numbers so that students could feel free not to use active workstations if they did not want to. All students and lecturers willing to participate in the study were included; there were no exclusion criteria.

Participation in the study was voluntary both for students and lecturers. It consisted of the first utilisation of active workstations for students during a lecture or, for lecturers, delivering a lecture in the active classroom. It also involved completing an online survey assessing the feasibility and acceptability of the setting for both students and lecturers. The students and lecturers were informed about the aim of the study, and they provided written consent before participation. The study was conducted in accordance with the Declaration of Helsinki and was approved by the University Ethics Committee.

**Fig. 1:**
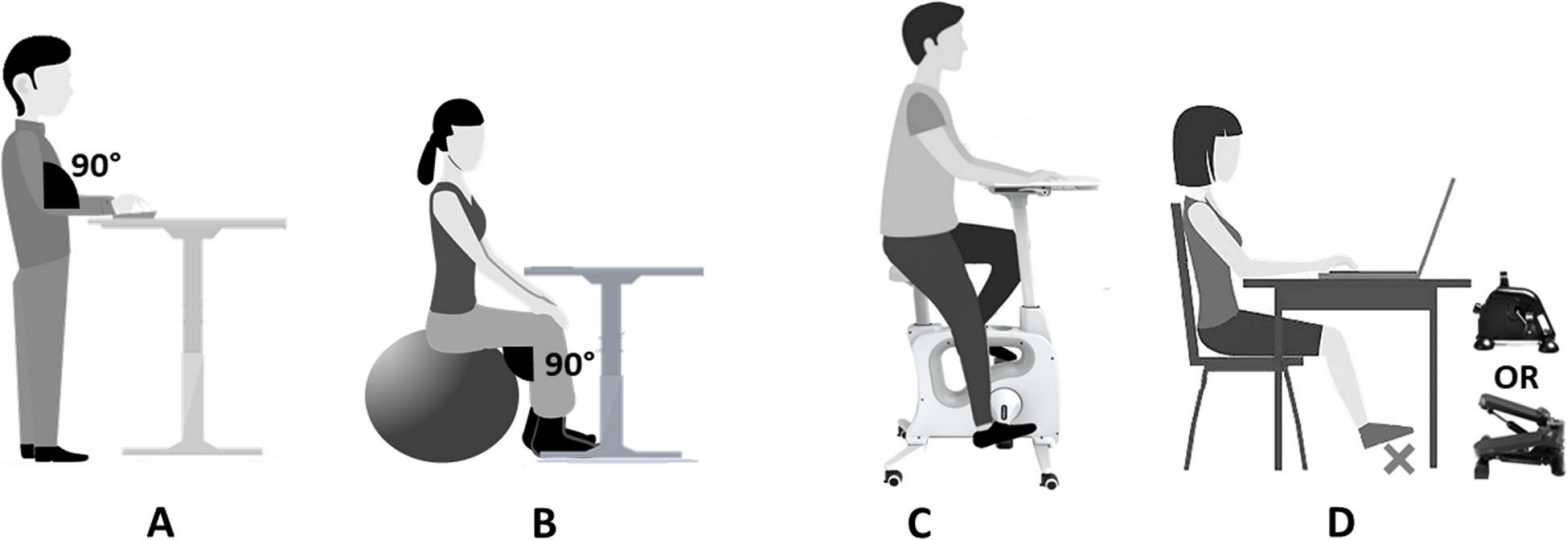
The four stations tested. **A** standing desk. **B** Swiss ball. **C** cycling-desks. **D** pedal- or stepper-board

At the beginning of each lecture, instructions were given for the proper use of each active workstation installed. A total of 4 types of workstations were available:

- *Six standing desks* (Skarsta, Ikea, Plaisir, France): desks at adjustable height were installed in the room (Fig. 1A). Recommended posture was: while standing, the desk level should be set up so that the elbows can rest on at 90°. Chairs were at disposal with those desks so that students were able to sit at any moment. It was recommended not to stay motionless but slightly and frequently change the support foot and/or fidget.
- *Six Swiss balls* (Domyos, Décathlon, Villeneuve-d’Ascq, France): three sizes of balls (S, M and L) were made available and height-adjustable desks installed (Fig. 1B). The recommendations were to sit on the ball in order to have the desk at the level of the sternum and hip and knee joints at 90°. Automated electrical inflators were also available, if necessary.
- *Six cycling desks* (Tek Active, Paris, France): specific desks mixed with ergometers were used for these type of stations (Fig. 1C). These cycling desks have saddles with adjustable height and a tablet with adjustable distance from chest. Students were instructed to adjust the saddle so that their legs were fully extended in the extreme position of the crankset. The tablet had to be adjusted so that student did not have to lean forward to write.
- *Six pedal- or stepper-boards* (Décathlon, Villeneuve-d’Ascq, France): portable cranksets and steppers were set up in front of traditional chairs (Fig. 1D). Height adjustable desks were also used for this station so that participants did not shock their knees while pedalling or stepping. Students were instructed to put the board on the floor in front of them in order to have their legs fully extended in the extreme position of the crankset.

It should be noted that additional active workstations could be quickly installed on demand. Yet, students had the choice to use or not active workstations and could thus choose the traditional workstations in first place. Similarly, further chairs and desks could also be installed on demand (a stock of chairs was available next to the entrance of the classroom).

For active workstations, instructions were written on cards fixed on the wall next to each station. At the beginning of each lecture, a 10-min setup procedure and equipment explanation was given, during which the cards were read, and students were free to ask any questions to lecturers, who were previously informed of the instructions. This 10-min period allowed students to adjust their workstation, if needed (the height of the standing desk, the height of the cycling desk seat, the cycling resistance, etc.). The selection of which type of workstation was left open, as it was important to test the spontaneous attractiveness of each active workstation. Each lecture lasted 2 consecutive hours, and students were encouraged to remain on the same active workstation during the whole duration of the lecture. At the end of each lecture, 15 min was allotted for students who had volunteered to answer an anonymous online survey on their phone or computer about their choice of active workstation. All the students who used active workstations agreed to answer the survey.

Separate surveys were created for students and lecturers. Both surveys tested the feasibility and acceptability of the active workstations and were completed at the end of the first utilisation (both for students and lecturers) of the active workstations. The student survey included 20 questions which focused on different aspects: i) demographic characteristics (self-reported age, weight, height, gender, level of education), ii) self-reported level of sporting activity (hours per week), and time spent sitting during the day, iii) type of active workstation used during the class (“standing desks”, “Swiss balls”, “cycling desks”, “pedal- or stepper-board”), (iv) estimation of the duration of use of the active workstation (during 2 h of classes), (v) subjective feelings about the use of the active workstation, both on physical aspects (activity, pain, fatigue, comfort), psycho-cognitive aspects (attention, boredom, anxiety/stress, participation in class, distraction (cell phone), restlessness) and academic aspects (comprehension of the lecture). Finally, the last part targeted the students’ intention to reuse these types of active workstations in future lectures. Questions on subjective feelings have been adapted from a study by Jerome et al. (2017), and students were asked to report whether the utilisation of active workstations during the lecture “decreased”, “increased” or “did not change” each aspect considered compared with a similar lecture attended using traditional workstations.

Lecturers were also asked to answer a similar survey (17 questions). Questions were about the same aspects as those previously described for students but from a teacher’s perspective. Therefore, questions focused on the following aspects: i) demographic characteristics (self-reported age, gender, level of education), ii) type of active workstations students used during the course (“standing desks”, “Swiss balls”, “cycling desks”, “pedal- or stepper-board”), iii) subjective observations about the students’ behaviour, particularly on psycho-cognitive aspects (attention, boredom, anxiety/stress, participation, distraction (cell phone), restlessness) and academic aspects (comprehension of the lecture). Finally, as for students, lecturers were asked about their willingness to reuse these types of active classrooms in future lectures.

### General project’s aim and educational component

Overall feasibility and student and lecturer favourability of implementing active workstations were determined from the responses to questions on the intention to reuse and the subjective feelings of students and lecturers.

This study is part of a more general policy of the university department set to reduce the sedentary behaviour of students. Therefore, using active workstations was explained to students after they experienced their first lecture in the active classroom. They were also given a booklet that explained the approach. Explanatory posters were also available in the hallway next to the “active room”. Therefore, the present study also contained an educational approach to raise awareness with students and lecturers about sedentary behaviours and solutions to reduce the associated risks.

### Data analysis

A period of 6 months was observed after the beginning of the academic year (active workstations were installed during the summer holidays) to collect sufficient responses to the survey, according to the students’ schedule. Data were first cleaned, then screened for duplication. Since they were asked to fill in their personal information, we were able to match survey responses to the list of registered students at the university to check for false profiles. It was not permissible to complete the survey twice with the same personal information.

Descriptive statistics were calculated for the general characteristics of the subjects (means and standard deviations) and the participants’ perceived activity and changes from traditional workstations (percentages).

## Results

### Students’ inquiry

#### The population

Survey responses from 663 students (62.9% men) have been included in the present study. The global characteristics of the participants are depicted in Table 1.

**Table 1:** General characteristics of the student population

	Mean
**Age (years)**	18.7 ± 1.6
**Weight (kg)**	65.5 ± 10.0
**Height (m)**	1.74 ± 0.09
**BMI (kg.m**^**−2**^**)**	21.6 ± 2.2

Data are mean ± SD

BMI: Body Mass Index

In terms of physical activity level, the participants were predominantly “active” (the recommendations for adults being 150 min of moderate activity/week). Indeed, 39.5% of participants reported engaging in more than 10 h of physical activity a week, and 49.8% between 5 and 10 h a week. Regarding time spent in sitting position, despite the high level of physical activity of the participants, 81.7% reported spending 4 h or more sitting in a day and 25% more than 7 h.

#### « active » lectures

**Fig. 2:**
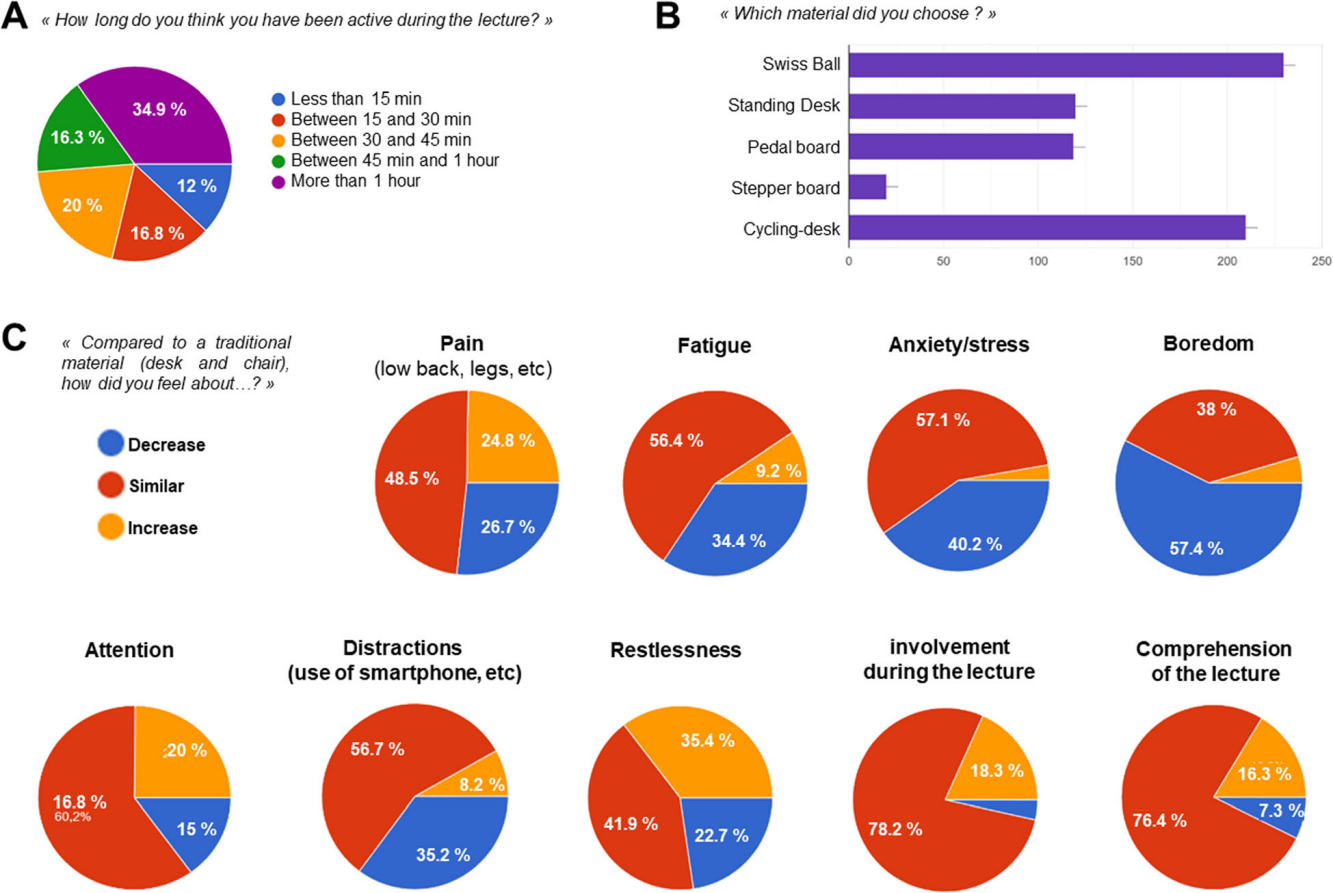
Graphic representation of student survey results regarding the acceptability and subjective markers of active workstations. *n* = 663. **A** perceived time spent active during the 2 h lecture. **B** Number of students per active workstation spontaneously chosen. **C** results of the survey regarding the different physical, psychosocial, and academic aspects investigated. Results are percentages

When asked how long they think they spent “active”, a third reported being active for more than an hour (Fig. 2A). In terms of active workstations, Swiss ball and cycling desks were the most spontaneously used (Fig. 2B).

Compared to a traditional lecture, 34.4% noted a decrease in their perceived fatigue, 40.2% a reduction in stress, and 57.4% admitted a decline in boredom with the active workstations (Fig. 2C). For 35.4% of the participants, there was an increase in restlessness during the lecture with the active workstations. Regarding more ‘academic’ parameters, a large majority of students did not feel any difference in attention and interactions with the teacher. However, distractions (use of mobile phone, social networks, etc.) seemed reduced for 35.2% of them.

**Fig. 3:**
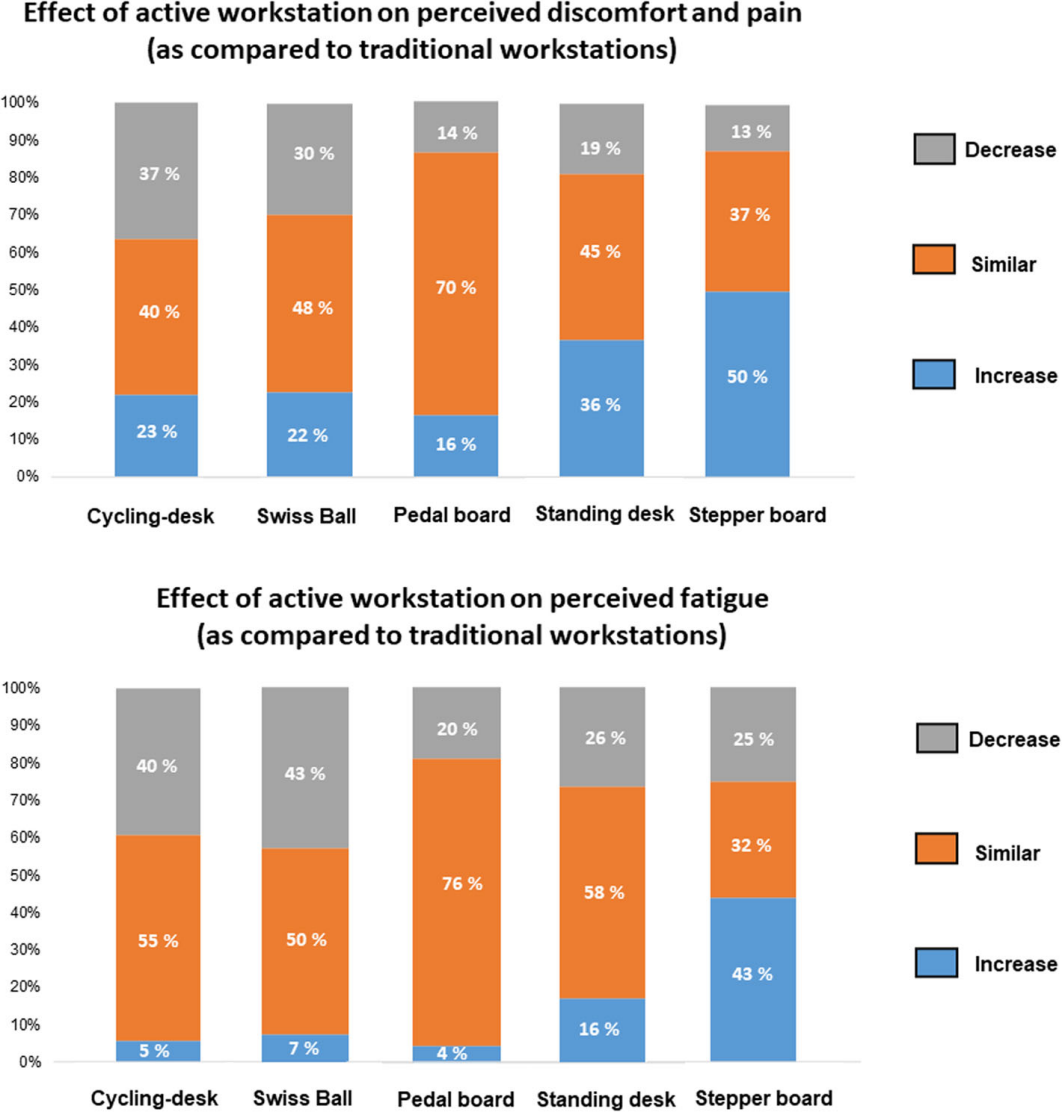
Effect of the different workstations on discomfort, pain and perceived fatigue. Each graph indicates the percentage of students who reported either a decrease (grey areas), similar (orange) or an increase (blue) in discomfort and pain and fatigue, over the total number of students who used each active workstation

When looking at the different tested workstations, the greater percentages of students who perceived a decrease in discomfort and pain compared with traditional workstation were observed with cycling-desk and Swiss Ball. Conversely, the greater percentage of students who perceived an increase in discomfort and pain compared with traditional workstation was observed with stepper board. Similar results were observed for perceived fatigue (Fig. 3).

Finally, 89.4% of students were in favour of using active workstations in future lectures if they are available.

### Lecturers’ inquiry

Fourteen teachers (8 men and 6 women) from the university answered the survey about their first experience in lecturing in the active classroom. They responded to questions on their perceptions of student restlessness, fatigue, boredom, stress, attention, participation, distraction and academic success. While some results were similar to the feelings of the students, some widely differed.

For example, while 35.4% of students perceived themselves to be more restless, only 21.4% of lecturers (4 out of 14) noted an increase in student restlessness compared to a regular lecture (50.0% no difference and 28.6% a decrease). 71.4% of lecturers (10 out of 14) felt that students were less tired at the end of the lecture than usual (28.6% no difference and 0.0% an increase of fatigue). 85.7% of them (12 out of 14) also felt that there was a decrease in boredom among students. In terms of students’ attention, lecturers’ results are more varied (35.7% found a decrease, 35.7% an increase and 28.6% no difference). A majority of lecturers (71.4%; 10 out of 14) found no effect of active workstations on students’ interaction during the lecture as compared to their usual lectures, while no one found a decrease. However, they were unanimous (100% of the lecturers) in the fact that active workstations reduced students’ distractions (chatting with other students, use of mobile phone, etc.).

Finally, 50% of lecturers felt that their lectures were not impacted in quality, while 35.7% found an increased quality and 14.3% a decrease. 78.6% of them (11 out of 14) felt that it had no particular impact on students’ ability to understand the lecture. 42.9% of them (6 out of 14) found the lecture more challenging to deliver because it required more attention and concentration. However, despite this difficulty, most lecturers (71.4%; 10 out of 14) are willing to use the classroom with active workstations again.

## Discussion

The present study determined the feasibility and acceptability of the implementation of active workstations in an ecological environment (i.e., classroom) in a French university. This research intended to collect first impressions and feelings of students and lecturers regarding the use of active workstations. Students and lecturers were broadly positive and favourable about such active workstations, although some pitfalls may have also been revealed.

First of all, it must be noted that the targeted population, students and lecturers at a sport university, are possibly more open-minded regarding physical activity and might be more likely to accept this type of experience. However, regularly practising sport does not prevent sedentary behaviours, which are considered as independent risk factors for cardiometabolic diseases regardless of physical activity level [4–6]. Indeed, regarding the sedentary time, despite the high level of physical activity of the participants, 81.7% reported spending 4 h or more sitting in a day and 25% more than 7 h. Thus, it seems crucial to modify the lifestyle habits associated with increased sedentary time (e.g., transportation, time at work, school, etc.).

For 35.4% of the participants, there was an increase in restlessness during the lecture with the active workstations. This can be explained by the “novelty” factor, and it should be investigated whether this phenomenon persists with an increase in familiarity with these types of active workstations. This can also explain the difficulty that lecturers reported facing when conducting their lecture. However, student and lecturer opinions were unanimous regarding a decrease in distractions, such as using a mobile phone or chatting with their peers. One could say that using active workstations can represent a distraction itself. Therefore, it seems important to observe a period of familiarisation with the active workstations in order to optimise their long term implementation.

Further, conducting an experiment on the effects of daily active workstation usage could bring clues regarding a reduction in restlessness. The objective of active workstations is not necessarily to increase attention or concentration, but at least not negatively impact it while interrupting sedentary behaviours and increasing the level of physical activity. The results of the present survey show that this goal appears to have been accomplished for most of the students and types of active workstations. In the long term, maintaining the standard model of passive sitting workstations contributes, in an involuntary way, to the alteration of the physical, psychological, social and cognitive health of students. Thus, it is crucial to modify this model and enable students to learn in conditions that promote their success in terms of their personal skills and know-how in the short, medium and long term. Indeed, by their status, students are the bridge between adolescence and the adult world and are therefore characterised as the adults of tomorrow with an educational, cultural and health legacy to pass on. Therefore, the university has a fundamental role to play in enabling the student to develop and succeed, but also to instill good working methods, and general lifestyles, for the rest of his or her life. Work habits acquired at school and during college are likely to persist during future employments. The actual situation seems at the exact opposite with sedentary behaviours commonly developed at school and college and persisting throughout the life cycle [28, 29]. Even worse, the daily sitting time has been shown to increase over the proceeding years of study [30]. Therefore, the objective of implementing this type of learning setup at the early stages of life is also relevant to educate the future workers on healthier working habits and overall life habits.

Regarding the preferred type of active workstations, cycling desks and Swiss balls seemed the most popular. They were also the ones that provided less perceived fatigue and discomfort while causing as little perturbation as possible on cognitive capacities. The fact that such active workstations allow users to remain sitting can explain such results since maintaining the standing position at standing desks tends to increase discomfort in the low back and legs. The pedal and stepper board was not used much, maybe because they were only portable devices placed on the floor, and the traditional chairs used were not ergonomically optimal to practice stepping compared to standing cycling. To optimise the implementation of any active workstations, it appears important to work beforehand on product ergonomy to adapt it to the specificity of the classroom environment. Nonetheless, one of the critical aspects regarding the implementation of such active workstations at a university remains the cost of purchasing large quantities of these devices. Even if cycling is optimal since it does not require servicing (like the Swiss ball needs regular reinflating) and is by far the most popular active workstation, it represents a substantial cost. In this regard, future research is necessary to objectively study the feasibility of options, notably in terms of cost-effectiveness and efficacy.

To that aim, while keeping the standing position may tend to increase fatigue and discomfort, simply allowing the students to break sitting times by standing on a spontaneous basis can appear an interesting option. When offered the opportunity to stand during lectures, it was shown that the total time spontaneously spent standing was on average of 7.2 min per hour, even after a familiarisation period of several days [31]. Therefore, although the use of specific active workstations, such as sit-to-stand desks, could increase this time by a little, this setup seems to provide a low amount of physical activity and does not appear to be the most optimal solution. However, this type of solution seemed to be extremely popular among students [16, 18]. In the present study, 34.9% of students reported being active more than an hour over the two hours lecture, much more than previously reported [31]. Although this could be attributed to the excitement of using such workstations for the first time, it should also be noted that the present population is composed of sport students, already familiarised with physical activity and more sensitive and aware of the importance of increased physical activity and decreased sedentary behaviours from a health perspective. Investigating the long-term use of such workstations on the sport students but also in the more inactive population can help generalise those results. However, and particularly for less active populations, standing requires voluntary action that could represent a break in attention and concentration and may not be automatised like pedalling, or maintaining balance on a Swiss ball.

Finally, 89.4% of students were in favour of using active workstations in future lectures if they had the possibility to, as well as 71% of lecturers, demonstrating the wide acceptance of such active workstations. This was in accordance with a previous investigation reporting a significant proportion of students (83%) and lecturers (87%) in favour of introducing sit-to-stand desks in the university classrooms of Midwestern university, USA [16]. Yet, in this previous study, only 2.8% of a total population of 993 interviewed students already used this type of active workstation, showing that this is far from being a commonly used tool. Considering the beneficial effect of active workstations on health by “breaking sedentary behaviours” which are major during academic work, it seems appropriate to continue the development of this type of pedagogy. However, in order to perpetuate and optimise it, it is necessary to understand the feelings on the “physical” and “restlessness” aspects and to modify or adapt, if necessary, the implementation. The ultimate challenge is to find the optimal amount of physical activity, which allows one to stay physically active while allocating sufficient cognitive resources to follow the lecture. The present results highlight the desire to continue utilising active workstations in students and lecturers, and the importance of supporting the implementation of a system to overcome the potential difficulties and to assist the changes in work habits.

One of the main limitations of the present study is the subjective evaluation. However, the present study focused on analysing the perceptions of students on a large scale, as well as lecturers’ perceptions, toward the acceptance of including active workstations in the classroom setup. Another important limitation of the present study, which limits generalising to all types of students, was the great homogeneity of the population tested, which were already widely physically active and open-minded to sport (sport university students). This study was a pilot study and thus a first step towards engaging a potentially more reluctant population, as it is known that some other populations of students are far less physically active [28]. Having the same investigation within a population of less active students could bring relatively different results.

## Conclusion

In conclusion, the first experience of active workstations in a university classroom was broadly positive and well accepted by students as well as lecturers. While noticing some difficulties at the beginning in adapting to an active workstation, the majority of participants were positive regarding the effects of such workstations on either their perceived fatigue, boredom or attention. The choice of the active workstation appears crucial to obtain an optimal increase in physical activity without deteriorating the quality of the learning, as well as the comfort of the students. Besides the cost, there is no unique and optimal solution. In fact, providing a variety of workstation types in an identical room, including traditional chairs and desks, might represent one of the best approaches. This would allow students to vary from one lecture to the next. Ultimately, it helps to individualise the process since each student may respond differently to the various types of workstations. It might also help to avoid boredom and a decrease of interest over time induced by the sustained usage of the same workstation. An assessment of the acceptance and spontaneous use of such active workstations over time is needed to see if long-term behaviour can be affected. This could be done by implementing a more longitudinal study also assessing sedentary periods outside the classroom.

Finally, a more in-depth analysis of cognitive abilities and energetic cost during lectures with such active workstations could objectively characterise those results. The feasibility of implementing such workstations in an ecological context appears achievable and depends on funding allocated to the project (e.g., choice of the type of the active workstations). In that sense, future research is needed to objectively determine the cost-effectiveness and efficacy of this learning model. In conclusion, the present study brings promising results toward a more general implementation of active workstations in French universities.

## Data Availability

The datasets used and analysed during the current study are available from the corresponding author on reasonable request.
